# Neonicotinoids and the Androgen Receptor: Structural Dynamics and Potential Signaling Disruption

**DOI:** 10.3390/biology15020126

**Published:** 2026-01-10

**Authors:** Mohd Amin Beg, Md Amjad Beg, Ummer Rashid Zargar, Torki Zughaibi, Adel Mohammad Abuzenadah, Ishfaq Ahmad Sheikh

**Affiliations:** 1 King Fahd Medical Research Center, King Abdulaziz University, Jeddah 21859, Saudi Arabia; taalzughaibi@kau.edu.sa (T.Z.); aabuzenadah@kau.edu.sa (A.M.A.); iasheikh@kau.edu.sa (I.A.S.); 2 Department of Medical Laboratory Sciences, Faculty of Applied Medical Sciences, King Abdulaziz University, Jeddah 21859, Saudi Arabia; 3 Department of Radiation Oncology, Rutgers Cancer Institute and Robert Wood Johnson Medical School, Rutgers University, New Brunswick, NJ 08901, USA; amjadbeg006@gmail.com; 4 Department of Zoology, Government Degree College, Anantnag 192211, India; uzssummer2@gmail.com; 5 King Faisal University, Hufof 36362, Saudi Arabia

**Keywords:** neonicotinoids, androgen receptor, molecular dynamics simulation, endocrine disruption, male reproductive problems

## Abstract

Neonicotinoids are synthetic nicotine-like compounds extensively used globally as insecticides for agricultural and urban purposes. Neonicotinoid-contaminated produce is a major public health concern worldwide. Limited epidemiological studies have shown an association of neonicotinoid exposure with abnormal semen analysis. This study aimed to elucidate the potential disruption of the androgen receptor (AR) by eight common neonicotinoids, including imidacloprid (IMI), acetamiprid, clothianidin, thiamethoxam, dinotefuran, thiacloprid (THI), nitenpyram, and nithiazine using docking and molecular dynamics (MD) simulation. The results showed good binding strength of all compounds (except THI) with AR, as indicated by high binding energy, high binding affinity, and number of bonding interactions. The results of MD simulation supported the conformational stability and structural dynamic behavior of the AR-IMI (receptor-neonicotinoid) complex upon binding. This was indicated by root mean square deviation showing stability of the complex; the root mean square fluctuation showing minimized residual fluctuations upon binding; the radius of gyration showing greater compactness of the protein structure; the solvent-accessible surface area showing no changes upon binding; and the Gibbs funnel energy of the landscape showing a stable conformation state with minimum energy and slight change in size and position of the sampled energy basin of the AR, with a stable equilibrium. Taken together, the structural dynamics results showed that neonicotinoids are bound stably in the same ligand-binding domain of the AR as the native ligand testosterone. This may perturb the natural binding of testosterone with the AR and potentially disrupt downstream signaling and biological pathways, leading to male reproductive dysfunction.

## Simple Summary

Neonicotinoids are chemicals structurally similar to nicotine and commonly used globally as insecticides for the prevention of insect-related losses on many vegetable and horticulture crops. Neonicotinoid sales constitute about a third of the global insecticide industry. Because of their extensive use, human exposure through neonicotinoid residue contamination of foods and the environment is a significant public health concern. The current study was conducted to assess androgen receptor disruption by these common neonicotinoid compounds and to predict their potential adverse effects on male reproductive functions. The study involved molecular modeling using molecular docking and molecular dynamics simulation.

## 1. Introduction

Neonicotinoids are chemicals with structural similarity to nicotine and are extensively used as insecticides because of their neuroactive properties against many crop-damaging insects [1,2,3,4,5,6,7,8]. According to the Food and Agriculture Organization [9], nearly one-third of the global agricultural food production is lost or wasted, with major losses occurring during initial agricultural production and postharvest handling and storage, largely due to pests. Many natural and synthetic pesticides have been used since the early 20th century to control these losses and increase crop production [2]. In 2021, insecticides accounted for about one-quarter of the 3.54 million metric tons of pesticides used worldwide, with neonicotinoids constituting approximately one-fourth of the insecticide industry [10,11,12]. Introduced commercially in 1991, neonicotinoids quickly replaced conventional insecticides, including organophosphates, carbamates, and pyrethroids, because of their broad insecticidal range, easy applicability, high water solubility, moderate resistance, low cost, high soil stability, effectiveness at low concentrations, relatively long half-life, systemic effectiveness, and presumed low acute toxicity to mammals, birds, and other higher organisms [4]. Neonicotinoids bind to nicotinic acetylcholine receptors in the central nervous system of insects, causing irreversible overstimulation and blockage of these receptors, resulting in paralysis and death [13]. Commonly used commercially available neonicotinoids include imidacloprid (IMI), acetamiprid (ACE), clothianidin (CLO), thiamethoxam (TXM), dinotefuran (DIN), thiacloprid (THI), nitenpyram (NIT), and nithiazine (TTNM), which are commonly used in agricultural and urban settings [14]. Neonicotinoids are used as agricultural insecticides for approximately 140 crop varieties, including grains, vegetables, and horticultural crops [14,15]. Four neonicotinoids (IMI, ACE, CLO, and TXM) account for more than 90% of the global neonicotinoid use [16,17]. In addition, neonicotinoids account for more than 80% of seed-applied insecticides [18].

The high water solubility and long soil retention of neonicotinoids promote runoff and environmental contamination, including surface and groundwater [17]. Less than 20% of seed-applied nicotinoids are absorbed by plant roots; the remainder accumulates in the soil and environment [5]. Owing to widespread use, neonicotinoids are now pervasive in many environmental compartments, and 81% of studies on neonicotinoid contamination in surface water reported maximum concentrations exceeding recommended thresholds [19]. From 2013 to 2022, water samples from 77 U.S. rivers showed imidacloprid in 44% of samples, with mean concentrations exceeding the chronic freshwater invertebrate benchmark by more than twofold [20]. Due to their extensive use, systemic action, and widespread environmental persistence, neonicotinoids pose exposure risks to non-target species, including wild insects, pollinators, aquatic species, birds, animals, and humans [1,21,22,23,24]. For example, adverse effects include colony collapse disorder in wild non-target insects; reduced bee population; disorganized nesting of bees; impaired reproduction and navigation in bumblebees and honeybees; and weight loss and disorientation in birds [3,5,18]. Adverse aquatic impacts include altered survival, growth, mobility, and behavior of aquatic insects and crustaceans, as well as declines in annual eel production in Lake Shinji, Japan, and uncoordinated movements in Nile tilapia [17,19,23,25,26]. Furthermore, experimental laboratory studies have shown adverse effects of neonicotinoid exposure, including genotoxic, neural, immune, endocrine, thyroid, renal, liver, testicular, and ovarian problems [24,27,28,29,30,31].

Human exposure to neonicotinoids through contaminated food and environmental residues is a growing public health concern [1,21,24,32,33]. In 2017, neonicotinoid residues, particularly IMI, were detected in 77% of fruits and vegetables in the United States [34]. Studies from China reported neonicotinoid contamination in 42.1–82.9% of fruits, vegetables, and tea samples [35]. In this regard, neonicotinoids and their metabolites have been detected in human urine, blood, serum, hair, breast milk, saliva, teeth, semen, bile, and cerebrospinal fluid [36]. About half of the US population aged ≥3 years has detectable levels of neonicotinoids, with higher exposure in children and Asians [37]. In China, neonicotinoids were detected in 91–97% of 196 paired urine and blood samples in young adults [38], and residues of ACE and IMI in all urine samples of pregnant women [39]. Epidemiological studies link human neonicotinoid exposure to oxidative stress, genotoxicity, and adverse neural, liver, and reproductive outcomes [5,22,32,40,41]. Neonicotinoid exposure is associated with adverse neurological outcomes, including autism spectrum disorder, memory loss, finger tremor, and heart developmental defects [24,42]. Limited reproductive and developmental studies revealed prenatal neonicotinoid exposure linked to decreased head circumferences and increased ponderal index in newborns, and fetal growth restriction [39,43]. Similarly, in utero exposure to ACE and IMI was associated with a high risk of cardiac septal defects [41]. Regarding reproductive problems, ACE, IMI, and CLO were detected in 98.4%, 86.5%, and 70.8% of seminal plasma samples from Chinese men, respectively, and were associated with decreased sperm progressive motility [40]. Residues of TXM and ACE were widely detected in serum, follicular fluid, and seminal plasma of couples seeking fertility treatment, and the presence of ACE in seminal plasma was associated with reduced embryo cleavage and lower biochemical and clinical pregnancy rates [44].

Animal studies demonstrated that neonicotinoid exposure is associated with reduced testicular weight, spermatogenesis dysregulation, and impaired semen quality in mice, rats, and other animals [24,45,46,47,48,49]. Exposure to IMI results in reproductive toxicity, including altered sexual behavior, reduced epididymal and seminal vesicle weight, reduced epididymal sperm concentrations, lower testosterone levels, increased abnormal sperm, and elevated sperm DNA damage in rats or mice [45,47,50,51,52]. In mice, IMI exposure impairs testicular histology and decreases androgen receptor (AR) expression [53]. Mitochondria are particularly important targets, as neonicotinoid exposure disrupts mitochondrial Ca^2+^ homeostasis, inhibits respiration, increases oxidative stress, leading to DNA damage, apoptosis, and lipid peroxidation [30]. The US Environmental Protection Agency reported that CLO, IMI, and TXM may adversely affect 67–79% of species and 56–83% of critical habitats in the United States [54]. In consideration of the ecological and potential human health effects, the European Union banned IMI, CLO, and TXM for all outdoor use in 2018 and withdrew the approval of THI in 2020 [55].

The literature review revealed limited studies on neonicotinoid exposure and human male reproductive effects, although several animal studies report adverse effects on male reproduction. Only one docking study was performed, which showed stable binding of IMI to mouse AR [53]. The present study aims to predict the potential endocrine-disrupting activity of eight commercially important neonicotinoid compounds against AR using molecular docking and molecular dynamics simulation.

Endocrine disruption by exogenous chemicals arises from interference with the synthesis, secretion, transport, metabolism, binding, action, or elimination of endogenous hormones [56,57]. Disruption occurs via multilayered mechanisms, including interactions with steroid hormone receptors and binding proteins, or modulation of enzymes, response elements, etc. [7,58,59]. Such disruptions are particularly critical at low exposure levels and during sensitive developmental windows, when hormonal signaling is tightly regulated. A principal mechanism involves direct interaction with hormone receptors, causing agonistic, antagonistic, or partial agonistic interference in signaling pathways essential for reproductive development and function [56,60]. The AR, a member of the steroid nuclear receptor superfamily, binds testosterone and dihydrotestosterone and is essential for male reproductive development, spermatogenesis, and broader male physiological functions [61,62]. Disruption of AR signaling can occur through both direct and indirect mechanisms. Direct interactions may involve agonistic or antagonistic binding to the AR ligand-binding domain, leading to altered receptor activation and dysregulated androgen-responsive gene transcription [63,64,65]. Indirectly, endocrine-disrupting compounds may influence androgen signaling by modulating steroidogenesis, hormone metabolism, or circulating androgen availability [65,66,67]. In addition, changes in AR expression, co-regulator recruitment, and non-genomic signaling pathways can further disrupt AR-mediated physiological processes [67]. One of the possible mechanisms proposed for AR-interfering compounds is that their binding may induce helix 12 displacement, leading to AF2 site distortions and impaired coactivator recruitment [68]. In this regard, binding of neonicotinoid compounds to AR may potentially disrupt native ligand–receptor interactions, leading to altered gene expression and impaired androgen-dependent processes, with implications for male reproduction.

Given the increasing global use of neonicotinoid insecticides, comprehensive in vivo, in vitro, epidemiological, and in silico studies are urgently needed to assess their potential risks to human and ecological health. In the present study, AR was selected as the target protein because AR signaling is a critical mechanism underlying male reproductive function [61,62]. The widespread and persistent use of neonicotinoids has led to substantial contamination of terrestrial and aquatic ecosystems worldwide. As summarized above, many laboratory and ecological studies demonstrated adverse effects of neonicotinoid exposure on male fertility, spermatozoa, and the male reproductive system, whereas human studies are limited. To address this knowledge gap, the present computational study aimed to investigate the interactions of eight widely used neonicotinoids with human AR using structural dynamics approaches. The findings provide mechanistic insights into neonicotinoid–AR interactions and their potential to disrupt AR activity, with implications for human male reproductive health.

## 2. Materials and Methods

### 2.1. Retrieval and Preparation of Receptor Protein and Ligands

**Figure 1:**
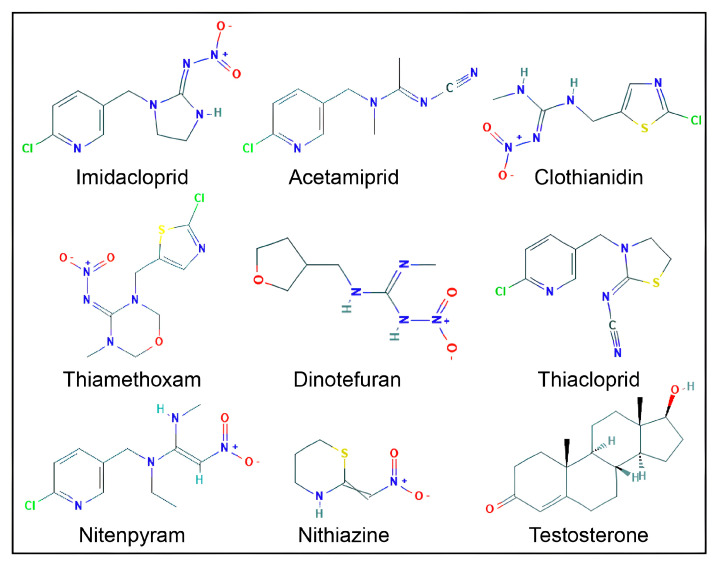
Two-dimensional structures of eight neonicotinoid compounds and testosterone. Oxygen (O, red), nitrogen (N, blue), chlorine (Cl, green), sulfur (S, yellow), and hydrogen (H, light grey) atoms are shown.

The co-crystalized three-dimensional (3D) protein structure of the AR ligand-binding domain was retrieved from the Protein Data Bank (PDB: https://www.rcsb.org; accessed on 20 January, 2024) as PDB ID 2AM9, with testosterone as the co-crystallized native ligand. The 3D structure of 2AM9 was viewed using Discovery Studio software (Discovery Studio Visualizer v19.1.0.18287; Biovia, San Diego, CA, USA). The two-dimensional (2D) structures of eight neonicotinoid compounds, IMI, ACE, CLO, TXM, DIN, THI, NIT, and TTNM, were retrieved from the PubChem database (https://pubchem.ncbi.nlm.nih.gov; accessed on January 20, 2024) and are illustrated in Figure 1. The Chemical Abstracts Service Registry Number (CAS No.), PubChem compound identities, and abbreviations for the neonicotinoid ligands are presented in Table 1. The downloaded SDF files were converted to PDBQT format, and all ligand structures were energy-minimized using the PyRx tool (PyRX-Python prescription 0.8) [69]. Illustration and analysis of the AR binding pocket studies were performed using the PyMOL graphic interface (The PyMOL Molecular Graphics System, Version 3.0 Schrödinger, LLC) [70]. All studies were conducted on a workstation with Windows 10, equipped with an Intel 10th-gen CPU, 12 GB of RAM, and high-end NVIDIA graphics.

### 2.2. Molecular Docking

The human AR ligand-binding domain was transformed from the PDB file format to the PDBQT format using MGL tools (version 1.5.7), according to a detailed protocol [71]. Docking poses were optimized using Discovery Studio. The AR active site was analyzed using ProteinsPlus (https://proteins.plus) web interface, which focuses on protein–ligand interactions and enables access to a wide array of functionalities [72]. Prior to docking, the native bound ligand (testosterone) was removed, water atoms, polar hydrogen atoms, and Kollman charges were appended, and the Gasteiger (-Marsili) charges were computed. Three-dimensional affinity grids were created at the geometric center of the target protein. The active site of the AR ligand-binding domain was enclosed in the grid box with a grid spacing of 1 Å for docking. The protein was kept stiff, whereas the ligands were completely flexible. AutoDock Vina 1.1.2 and InstaDock v1.0 (incorporating Quick-Vina-W), which utilize a similar hybrid scoring function, were used for the docking of eight experimental neonicotinoid ligands with the target protein 2AM9 (ligand binding domain of AR). The details of the software and methodology were described [73]. The binding free energy between the ligand and the protein was calculated using the hybrid scoring function of the software. Docking runs were initiated from the command prompt after identifying the binding site and preparing the receptor ligand [74].

### 2.3. Protein-Ligand Interactions

Protein-ligand interactions were investigated using a PDBQT output file of the docking complex. Structural and receptor–ligand interactions were analyzed and validated using Discovery Studio 2019. This software generated a binding analysis of a 2D interpretation of interacting residues from the interfaces of high-quality protein and ligand contacts. The results provided an instructive depiction of intermolecular interactions and their strengths, including hydrogen bonds, hydrophobic contacts, and atom accessibility.

### 2.4. Binding Energy, Binding Affinity, and Ligand Efficiency

For each neonicotinoid compound, the binding energy, binding affinity (pKi), and ligand efficiency were derived from the InstaDock (v1.0) docking scores (ΔG values), using the equations as applied in the InstaDock method [73]. The pKi is a measure of binding affinity and is the negative logarithm of the affinity constant (Ki). A higher pKi value corresponds to a lower Ki value and indicates a greater binding affinity of a compound to a receptor, resulting in a more potent pharmacological effect [75]. The pKi values were generated by the docking software InstaDock [73], which integrates calculations from the ∆*G* parameter utilizing the following formula:

∆*G* = RT(Ln *Ki*_pred_)

*Ki*_pred_ = e^(∆G/RT)^

p*Ki* = −log(*Ki*_pred_)

where ∆*G* is the binding affinity (kcal mol^−1^), R (gas constant) is 1.98 cal*(mol*K)^−1^, T (room temperature) is 298.15 Kelvin, and *Ki*_pred_ is the predicted affinity constant. Ligand efficiency (LE) is a commonly applied parameter for selecting favorable ligands by comparing the values of average binding energy per atom. The following formula was applied to calculate LE:

LE = −∆*G*/N

where LE is the ligand efficiency (kcal mol^−1^ non-H atom^−1^), ∆*G* is the binding affinity (kcal mol^−1^), and N is the number of non-hydrogen atoms in the ligand.

### 2.5. Binding-Pose Comparison Analyses

The docking poses of the neonicotinoid ligands and the native ligand testosterone were compared to ensure that the neonicotinoid ligands were bound to the same AR ligand-binding pocket as the testosterone. Furthermore, within the binding site, the interacting residues common to neonicotinoid compounds and testosterone were compared.

### 2.6. Molecular Dynamics (MD) Simulations

Molecular dynamics simulations predict the conformational stability and dynamic behavior of the protein-ligand complex, providing information on the ligand’s interaction with the protein and changes in the complex over time [76]. MD simulations were performed using GROMACS 5.1.4 with analysis tools (gmx rms, gmx rmsf, gmx gyrate, gmx sasa, gmx, and hbond) installed on a multi-core Linux workstation (Intel Xeon CPU, 64 GB RAM). The GROMOS-96 force field 43a2 was used to determine dynamic behavior within given sets of physicochemical conditions as previously described [77]; for in silico studies, different calculations were performed for proteins and ligands. Using GROMACS trajectory tools (gmx, trjconv, and gmx select) along with text-filtering utilities, the ligand files present in the docked files were separated into individual files independent of their respective complex files. The PRODRG server was used to build the topology and other parameters of the IMI, as previously described [78]. While widely used for rapid topology generation, PRODRG relies on default partial charges and parameter assignments that may be less optimal for highly quantitative free-energy estimations compared with recent small-molecule force-field parameterization frameworks (e.g., CGenFF-, ACPYPE-, or Antechamber-based workflows). Accordingly, the present findings are interpreted in a comparative and mechanistic context rather than as absolute free-energy estimates. The generated topology files of AR and IMI were merged. Additional ligand atom information and other parameters were included in the system topology files for further steps, as previously described [79]. The water model SPC216 was used to dissolve the system (AR protein) and complex (AR-IMI) to determine AR stability, as previously described [80]. As a control, MD simulation was performed in water at 298 K (temperature) using a Langevin thermostat (an algorithm for maintaining the temperature in MD simulation) with a collision frequency of 1.0 ps^−1^ and pressure of 1 atm, using a Berendsen barostat (an algorithm for controlling the pressure in MD simulation) with a relaxation time of 2.0 ps. All atoms of the macromolecules, the AR and AR–IMI complex, were equilibrated in a three-dimensional box with a buffer distance of approximately 10.5 Å between the protein surface and the simulation box boundaries on all sides. The AR and AR–IMI complex were immersed in water and well equilibrated; overlapping molecules were deleted, with all atoms well confined within the cubic box. All bonds involving hydrogen atoms were constrained using the SHAKE algorithm, allowing a 2-fs integration time step. All the systems were prepared in a defined box; the AR was placed in the center of the box and padded with water. In each system, the potential energy was minimized using the steepest descent-based algorithm, and all bad contacts were removed up to a threshold tolerance of 1000 kJ/mol. NaCl was added at cationic and anionic concentrations to neutralize the overall charge of the system. These details are presented in the NPT, NVT, and MD parameter files. Physicochemical conditions play a vital role in conformational and structural changes. The system was ready to run after the completion of preliminary preparations. After the completion of the production run, trajectory files were generated for further analysis. The trajectories were generated in the form of binary files, including gmx rms, gmx gyrate, gmx rmsf, gmx hbond, gmx sasa, and the Gibbs funnel energy of landscape (FEL), using GROMACS 5.1.4. PyMol and Visual Molecular Dynamics were used to prepare a graphical representation of the 3D models and for structure visualization [81]. The results of MD simulations were presented as the average potential energy of the system, root mean square deviation (RMSD), root mean square fluctuation (RMSF), radius of gyration (Rg), solvent-accessible surface area (SASA), hydrogen bond dynamics, and FEL.

## 3. Results

### 3.1. Target Protein Sequence

The 3D structure of the human AR ligand-binding domain contains 250 of the 919 amino acid residues of a full-length AR protein. The AR protein has three functional domains: an amino-terminal domain, a DNA-binding domain, and a carboxyl- or ligand-binding domain. Protein–ligand interactions and structural features of the androgen receptor (AR) ligand-binding pocket were examined using the ProteinsPlus web interface [72]. The AR active site consisted of the following amino-acid residues: Leu-701, Leu-704, Asn-705, Glu-706, Leu-707, Gly-708, Glu-709, Gln-711, Trp-741, Met-742, Met-745, Val-746, Ala-748, Met-749, Arg-752, Tyr-763, Phe-764, Ala-765, Met-780, Met-787, Leu-873, Phe-876, Thr-877, Phe-878, Leu-880, Val-889, Phe-891, Met-895, and Ile-899.

### 3.2. Molecular Docking

**Figure 2:**
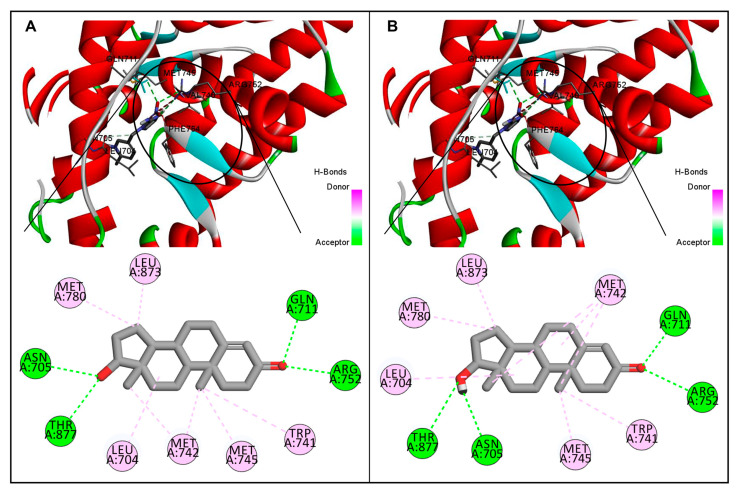
Comparative illustration of native interaction and re-docking poses of the androgen receptor and testosterone. (**A**): Interaction pose showing native bound ligand testosterone in the ligand binding pocket of the androgen receptor and the interacting amino-acid residues. (**B**): Re-docked pose of testosterone in the ligand binding pocket of the androgen receptor and the interacting amino-acid residues. For both poses, residues interacting with a hydrogen bond are shown in green, and those with a pi-alkyl bond are shown in pink. Note that the native-bound and re-docked testosterone were bound in the same active site of the androgen receptor with identical interacting residues for both interaction poses.

**Figure 3:**
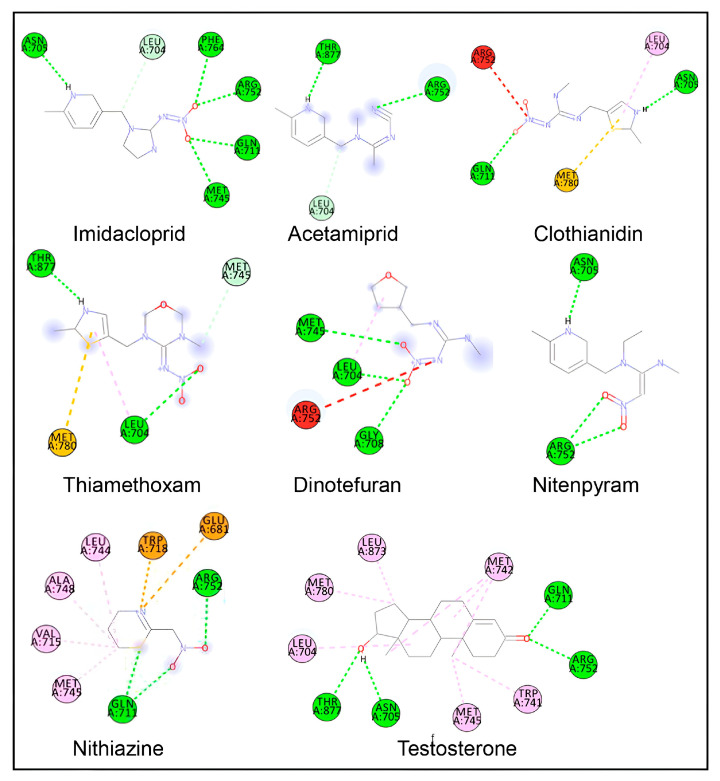
Two-dimensional ligand–protein interaction plots of various neonicotinoid ligands during molecular docking in the ligand-binding site of the androgen receptor. Amino-acid residue interactions of the native ligand of the androgen receptor (testosterone) are also shown. Amino-acid residues with hydrogen bonds are shown in green color, pi-alkyl bonds in light pink color, pi-sulfur bonds in light yellow color, pi-charge bonds in deep yellow color, and carbon-hydrogen bonds in light pink color. Amino-acid residues with unfavorable interactions are in red.

**Figure 4:**
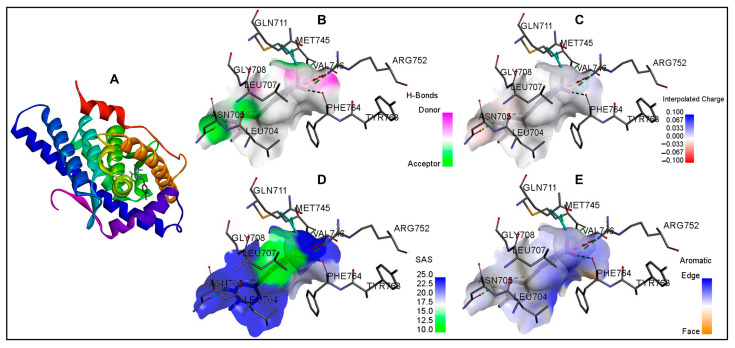
Molecular docking of imidacloprid in the ligand-binding site of the androgen receptor is shown in ribbon representation and surface representations. (**A**): ribbon representation; (**B**): surface representation with pink and green surface of the binding site illustrating the donor and acceptor hydrogen bond interaction regions; (**C**): surface representation with binding pocket surface color-coded according to interpolated charge (blue/red = positive/negative), (**D**): surface representation with solvent accessibility surface showing the residues from blue for exposed to green for buried; and (**E**): surface representation with surface colored blue for aromatic ring edges and orange for aromatic ring faces.

The basic orientation between the AR ligand-binding domain and neonicotinoid ligands was established using InstaDock by setting the grid parameters in the CONF file for the active-site-binding pocket. The orientations of the binding site-prepared grid parameters were center X (25.027), Y (6.887), and Z (4.24), and size X (20), Y (22), and Z (20). In the first step, the binding site of the AR-testosterone native complex was confirmed by redocking AR with the native ligand, testosterone. The interacting residues for the native-bound and redocked testosterone matched exactly in the same active site, which confirmed that the bound ligand grid parameters were satisfactory (Figure 2; Table 2). In the second step, AR was docked with eight indicated neonicotinoids: IMI, ACE, CLO, TXM, DIN, THI, NIT, and TTNM. The interaction binding poses of the neonicotinoid ligands, and the AR ligand-binding pocket are shown in Figure 3. Table 3 shows the data for predicted binding energy, binding affinity (pKi), and ligand efficiency, and Table 4 shows the data on molecular interactions and hydrogen bonds. The analysis of binding strength scores and interaction analysis of the AR-neonicotinoid docking complexes showed that seven of the eight neonicotinoid ligands were bound within the same binding site of the AR as the native ligand testosterone. IMI had the highest docking score among all neonicotinoid compounds, with a docking score of −8.0 kcal/mol, followed by NIT, TXM, ACE, CLO, DIN, and TTNM (Figure 3; Table 3). However, THI did not bind to the same active site and did not interact with the AR. Instead, THI showed a high negative value for the binding energy against AR. Further analysis of other parameters for this neonicotinoid was not performed. Analysis of the docking complexes of seven neonicotinoid ligands with the AR showed similar binding poses to the native ligand testosterone, thus validating the docking accuracy (Figure 3). To gain insight into the interactions between the neonicotinoid compound and AR, the docking of IMI with the AR protein was illustrated using surface visualization (Figure 4). Interactions in the binding plot were further analyzed using different surface features of the IMI–AR complex, including hydrogen bonds, charge, hydrophobicity of receptor residues, and aromatic rings. These surface plots of the IMI–AR complex further visualized the donor and acceptor hydrogen atoms, interpolated atomic charge, solvent accessibility, and ionizability (Figure 4).

Docking interaction analysis showed that IMI interacted with six amino-acid residues, Leu-704, Asn-705, Gln-711, Met-745, Arg-752, and Phe-764, in the AR ligand binding site with six hydrogen bonding interactions (Figure 3; Table 4). Similarly, ACE interacted with three amino acids (Leu-704, Arg-752, and Thr-877) in the AR ligand-binding site with three hydrogen bond interactions, and CLO interacted with five amino acids (Leu-704, Asn-705, Gln-711, Arg-752, and Met-780) in the AR ligand-binding site with two hydrogen bonds, one pi-alkyl, and one pi-sulfur interaction. Furthermore, TXM interacted with four amino acids (Leu-704, Met-745, Met-780, and Thr-877) in the AR ligand-binding site via three hydrogen bonds and one pi-sulfur interaction; DIN interacted with four amino acids (Leu-704, Gly-708, Met-745, and Arg-752) via three hydrogen bond interactions; NIT interacted with two amino acids (Asn-705 and Arg-752) in the AR ligand-binding site via two hydrogen bond interactions; and TTNM interacted with eight amino acids (Glu-681, Gln-711, Val-715, Trp-718, Leu-744, Met-745, Ala-748, and Arg-752) via two hydrogen bond, four pi-alkyl, and two pi-charge interactions.

For the bound native ligand testosterone, 10 amino-acid residues in the AR ligand-binding site (Leu-704, Asn-705, Gln-711, Trp-741, Met-742, Met-745, Arg-752, Met-780, Leu-873, and Thr-877) were involved in the interactions with four hydrogen bonds and six pi-alkyl interactions (Figure 3; Table 4). A comparison of the AR amino-acid residues interacting with testosterone and those interacting with neonicotinoid ligands showed a commonality of 83% (five of six amino-acid residues matching) for IMI. Furthermore, 100% commonality (all amino-acid residues matching) was found for the AR interacting residues with ACE, CLO, TXM, and NIT, whereas 75% and 38% commonalities were, respectively, observed for DIN and TTNM (three of four and three of eight amino-acid residue matching, respectively).

### 3.3. Molecular Dynamics (MD) Simulations

Time evolution analyses were monitored for MD simulation (at 298 K) of the top-scoring neonicotinoid, IMI, in complex with AR. The evaluated time evolution plots included RMSD, RMSF, Rg, SASA, and FEL for both the native AR protein and the AR-IMI docking complex. The corresponding results are presented below.

#### 3.3.1. Average Potential Energy of the System

The average potential energy of AR and its docking complex with IMI (AR-IMI) was monitored at a constant temperature of 298 K and pH 7.0 to determine the equilibration point of the systems before the MD simulation production run. The data is shown in Table 5. Both systems showed initial fluctuations in potential energy followed by stabilization at distinct average values, with the AR–IMI complex exhibiting a more negative mean potential energy, indicative of enhanced stability upon ligand binding.

#### 3.3.2. Root Mean Square Deviation (RMSD)

**Figure 5:**
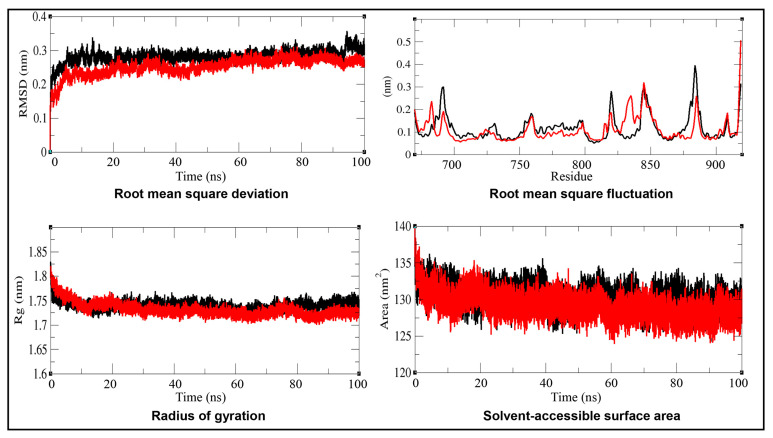
Molecular dynamics simulation analyses of the androgen receptor (AR; in black) and the AR–imidacloprid (AR–IMI; in red) complex over a 100 ns simulation period at 298 K. Time-evolution plots depict root mean square deviation (RMSD), root mean square fluctuation (RMSF), radius of gyration (Rg), and solvent-accessible surface area (SASA). Comparative analyses indicate enhanced structural stability and compactness of AR upon IMI binding.

The RMSD was calculated for the alpha carbon (Cα) backbone to assess the structural stability of AR relative to its experimental conformation. The RMSD results shown in Figure 5 and Table 5 showed similar fluctuations for both the native AR and the AR–IMI complex throughout most of the simulation time frame (up to 90 ns). However, after 90 ns, the AR–IMI complex exhibited reduced fluctuations compared with the native protein, indicating enhanced structural stability upon ligand binding. The lower average RMSD values of the AR–IMI complex compared to native AR further confirm increased conformational stability of the receptor upon IMI binding.

#### 3.3.3. Root Mean Square Fluctuation (RMSF)

The RMSF of the AR and the AR–IMI complex was analyzed as a function of residue number to evaluate residue-level flexibility upon ligand binding, as shown in Figure 5. The RMSF plot revealed localized fluctuations in several regions of the protein or system. These fluctuations were minimized after the binding of IMI to the AR, indicating stabilization of flexible regions.

#### 3.3.4. Radius of Gyration (Rg)

The Rg values for the AR and AR–IMI complex are presented in Figure 5 and Table 5. The Rg plot indicated that the native AR adopted a slightly less compact conformation compared with the AR–IMI complex, as reflected by the lower average Rg value of the complex. Minor fluctuations observed in the Rg of the AR–IMI complex are consistent with localized residue flexibility revealed by the RMSF analysis, indicating subtle conformational adjustments without loss of overall structural compactness.

#### 3.3.5. Solvent Accessible Surface Area (SASA)

The SASA values of the AR and the AR–IMI complex were calculated from the MD simulation trajectories, and the data are shown in Figure 5 and Table 5. The SASA plots indicated no changes in the SASA values of either the native AR or the AR–IMI complex over the 100 ns simulation time, suggesting overall structural stability. Notably, a slight decrease in the average SASA value of the AR–IMI complex was observed, indicative of enhanced molecular packing upon ligand binding. This observation is consistent with the Rg analysis.

#### 3.3.6. Hydrogen Bond Dynamics

**Figure 6:**
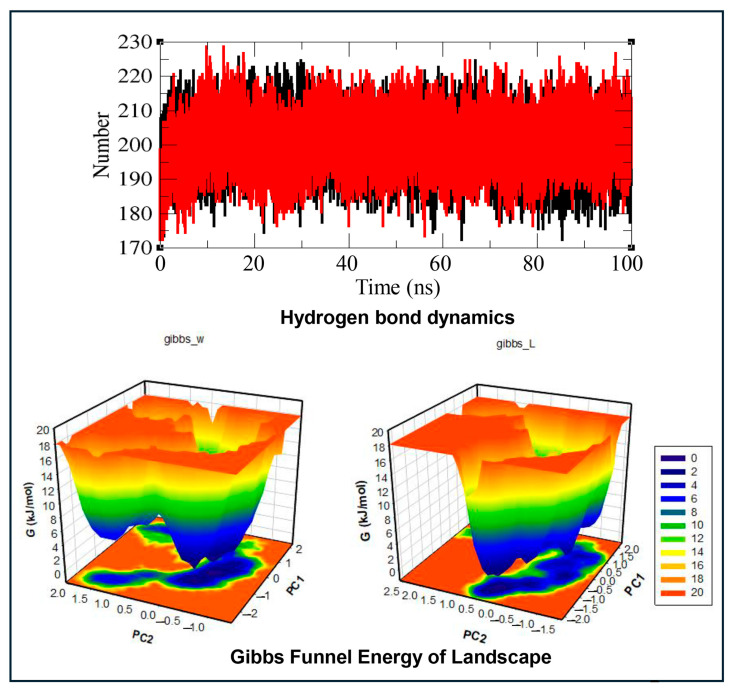
Molecular dynamics simulation analysis of androgen receptor (AR) and the AR–imidacloprid (AR–IMI) complex over a 100 ns simulation period. The top panel shows time evolution of intramolecular hydrogen bond dynamics (native AR is shown in black and the AR–IMI complex in red). The lower panel shows Gibbs funnel energy of landscape (FEL) plots constructed using the first two principal eigenvectors, illustrating the FEL of the native AR (left) and the AR–IMI complex (right). Deep blue regions correspond to global energy minima, indicating stable conformational states. The FEL analysis demonstrates that IMI binding subtly alters the size and spatial distribution of the energy basin while preserving a single stable global minimum, supporting a stable AR–IMI complex.

Hydrogen bond analysis of the AR–IMI docking complex was performed to determine the impact of the ligand on the protein structure during MD simulation within the 100 ns time frame. The hydrogen bonds were monitored with respect to time evolution using a distance cutoff of 3.5 Å. The data for the time evolution plots for hydrogen bond dynamics analysis of the native AR and the AR–IMI complex within the 100 ns time frame of MD simulation are shown in Figure 6 and Table 5. The AR–IMI complex showed a slightly higher average hydrogen-bond count than native AR, indicating preserved hydrogen-bonding networks and supporting overall complex stability.

#### 3.3.7. Gibbs Funnel Energy of Landscape (FEL)

In the present study, the FEL was analyzed to visualize the lowest energy minima and spatial conformational landscape of the AR and AR–IMI protein complex using the first two eigenvectors (EV1 and EV2), thereby providing further insight into AR conformational behavior. The FEL results are presented in Figure 6, where the FEL is represented by a deep blue color and is the global minimum with the lowest energy state (i.e., the most stable conformational state). The contoured graph of the FEL of the AR and AR–IMI complex depicted a deeper blue color, indicating the most stable conformation state with minimum energy. The FEL plots implied that upon IMI binding with AR, subtle changes were observed in the size and spatial distribution of the energy funnel, within a single stable local global minimum. The FEL graph of the native protein showed only a single global minimum that was bound to a single basin. Similarly, during IMI binding to AR, the complex acquired different spatial conformational states; however, it remained a single deep blue global minimum with a wider basin compared to AR with different types of populations. These findings suggest that IMI binding with AR slightly modulates the conformational landscape of AR without disrupting its overall stability.

## 4. Discussion

Pervasive environmental contamination with neonicotinoids raises significant concerns for human health [1,7,8,22,24,32,33,36]. The present study investigated the structural interactions of eight widely used neonicotinoids, IMI, ACE, CLO, TXM, DIN, THI, NIT, and TTNM, with the human AR ligand binding domain (PDB ID: 2AM9) using molecular docking and MD simulations. The redocking of AR with the native ligand testosterone reproduced binding energy and interacting amino acid residue results similar to those of the native interaction (AR and testosterone), which confirmed and verified the accuracy of molecular docking. Docking analysis showed that seven of the eight neonicotinoids (IMI, ACE, CLO, TXM, DIN, NIT, and TTNM) exhibited good binding interactions with AR, as reflected by their binding free energy and binding affinity (pKi values). One ligand, THI, did not bind to the AR ligand-binding domain and was therefore excluded from further analysis. This lack of interaction of THI may be attributed to its structural uniqueness, particularly the presence of a cyanomidine substituent on the cyclic moiety, which differentiates it from the structurally related ACE that possesses an acyclic moiety. This cyano-functional group on the cyclic moiety may introduce steric hindrance or disfavor electrostatic interactions, preventing stable accommodation within the AR ligand-binding pocket. The absolute values of the binding energies revealed comparable binding strengths among the seven ligands for AR, with IMI exhibiting the highest binding free energy and affinity, ranking it as the top-scoring compound among the tested neonicotinoid ligands. In addition to binding strength, complete overlap of interacting residues between the testosterone and several neonicotinoids (ACE, CLO, TXM, and NIT) further supported stable docking interactions. Moreover, this was also observed for IMI and DIN, in which all amino-acid residues, except one, matched the interacting amino-acid residues for testosterone. Furthermore, conventional hydrogen bond interactions for all neonicotinoid ligands, together with hydrophobic pi-alkyl bonds within the AR ligand-binding domain, reinforced favorable binding. The variations in binding scores and interactions among neonicotinoids likely reflect subtle structural differences. For example, IMI, ACE, and NIT share a common 6-chloro-3-pyridine methyl scaffold but have minor variations in substituents, such as nitroguanidine, cyanomidine, and nitromethylene attached to the scaffold, influencing interaction patterns and binding to the AR. Similarly, minor structural differences between TXM and CLO, differing only in the CH_2_OCH_2_ grouping [82], apparently lead to differences in binding affinity. Notably, CLO was first discovered as a prominent TXM metabolite in cotton plants [83]. However, DIN and TTNM have major differences in their structures compared to other neonicotinoid compounds, which probably contributed to their low binding scores. The top-scoring neonicotinoid IMI with the highest binding energy, binding affinity, and number of hydrogen bonds was selected for further evaluation of the stability of the AR–IMI docking complex by MD simulation.

The MD simulation parameters, such as time evolution analyses for RMSD, RMSF, Rg, SASA, and FEL, collectively supported the conformational stability and favorable dynamic behavior of the AR–IMI docking complex. Overall, the fluctuations in the average potential energy of AR and the AR–IMI complex remained minimal, indicating a stable and well-equilibrated MD simulation. The lower value for average potential energy for the AR–IMI complex was indicative of enhanced stability upon ligand binding. Ligand binding can perturb receptor structure; thus, RMSD was used to assess temporal deviations from initial conformation. The RMSD profiles demonstrated that both AR alone and AR–IMI complex remained stable throughout the simulation, with no major structural deviations observed until 90 ns. Similarly, residue-level flexibility was assessed using RMSF, which measures average positional deviations of individual residues over time from a reference position. The RMSF results showed reduced residual fluctuations upon IMI binding to AR, further confirming the stabilization of the AR structure in the complex. Furthermore, Rg analysis reflects the distribution of atoms of a protein around its center of mass and provides insight into the protein’s tertiary structure compactness. The lower Rg values for AR–IMI complex compared with AR alone indicated a more compact and structurally stable conformation upon ligand binding. In addition, the SASA of a protein is related to the surface area of the protein accessible to the solvent, and an increase in SASA indicates that the protein is in a more unfolded state, thus providing insights into the folding/unfolding behavior and stability of proteins [84]. No changes in the SASA of the AR and AR–IMI complex throughout the simulation time of 100 ns suggested a stable complex. A slight decrease in the average SASA value of the AR–IMI complex suggested decreased unfolding propensity and enhanced structural integrity. Hydrogen bond dynamics, a key intermolecular interaction contributing to protein stability, alongside salt bridges and van der Waals forces [85], showed comparable values for the native AR and AR–IMI complex throughout the 100 ns MD simulation, supporting the stability of the complex. A slightly higher average hydrogen-bond count for the AR–IMI complex than native AR further supported overall complex stability. FEL analysis further illustrated the protein folding behavior and identified the most stable conformational ensemble. IMI binding with the AR subtly altered the size and position of the AR energy basin while maintaining a stable equilibrium state. Collectively, molecular docking analysis parameters, including binding energy, binding affinity, interacting amino acid residues, and hydrogen bond interactions, together with MD simulation analyses, indicated that neonicotinoids stably occupied the same ligand-binding domain of the AR as the native ligand testosterone. Consistent with the mechanisms proposed for other AR-interfering compounds, neonicotinoid binding may induce helix 12 displacement, leading to AF2 site distortions and impaired coactivator recruitment [68]. Such interference could disrupt normal androgen binding, resulting in dysfunction of AR signaling.

A major concern is that endogenous hormones and synthetic AR agonists typically exhibit binding free energies comparable to testosterone (≈ −11 kcal/mol), which are associated with full receptor activation, whereas slightly weaker values of −8 to −12 kcal/mol are associated with antagonists and agonist/antagonistic chemicals [64]. In this regard, the binding energies observed for neonicotinoids (≈ −8 kcal/mol or lower) are more consistent with partial, transient, or modulatory interactions or weak antagonistic activity. Nevertheless, many environmental endocrine disruptors exert biological effects despite low receptor affinity, through partial receptor engagement, competitive interference with endogenous ligands, or altered receptor dynamics and co-regulator recruitment [56,57].

Previous molecular docking studies of neonicotinoids have primarily focused on nicotinic acetylcholine receptors in insects and humans [86,87,88]. In silico studies targeting human AR are lacking, except for one study, which examined IMI interactions with the mouse AR ligand-binding domain (PDB ID: 2QPY) [53]. The reported study demonstrated stable IMI binding at the AR active site, supported by RMSD stability, hydrogen bonding, and interactions with key ligand binding residues. Overall, these findings are consistent with our results. However, direct comparison of residue-level interactions is not feasible due to species differences between mouse and human AR and the exclusion of other neonicotinoids in the reported study.

Epidemiological evidence linking neonicotinoid exposure to adverse effects on human health, especially reproductive health, remains limited. Although direct comparisons are not possible, available studies support the implications of our in silico findings regarding the potential adverse effects of neonicotinoids on male reproductive function. A summary of reported reproductive effects of various nicotinoids is provided in Supplementary Table S1. Briefly, higher levels of IMI and its metabolite, 6-chloronicotinic acid, in the semen and blood of farm workers were associated with lower sperm concentrations, motility, and normal sperm morphology [50]. In male farm workers from Thailand, five neonicotinoid compounds and their metabolites were detected in 60% of urine samples [89]. Significant associations were found between urinary neonicotinoid levels and serum hormone concentrations: a positive association between IMI and testosterone, dehydrocorticosterone (DHC), and dehydroepiandrosterone (DHEA); a positive association of IMI-olefin (IMI metabolite) and DHEA; a negative association of TXM with DHC and deoxycorticosterone; a positive association of TXM, CLO, and N-desmethyl-ACE (ACE metabolite) with androstenedione; and a negative association of CLO and TXM with cortisone. All of these suggest deregulation of androgen and other hormones. Furthermore, metabolites IMI-olefin, N-desmethyl-ACE, and DM-CLO (CLO metabolite) were detected in 71–98% of human seminal plasma samples, with IMI-olefin associated with lower total and progressive sperm motility [40]. In China, 10 neonicotinoids, including seven compounds evaluated in the present study, were detected in 72–100% of urine samples of boys and girls (median age, 13.7 years) [90]. Higher urinary THI concentrations were positively correlated with delayed genital development in boys and early axillary hair development in girls, whereas total neonicotinoid concentrations were negatively associated with the genitalia-development stage in boys. In the US National Health and Nutrition Examination Survey (2015–2016) cohort of male population aged 6 years or older, serum total testosterone was 38%, 21%, and 25% lower, with a 10-fold increase in urinary total neonicotinoids, 5-hydroxy-imidacloprid, and N-desmethyl-acetamiprid, respectively [91].

Experimental studies in laboratory species, including rats and mice, demonstrated that neonicotinoid exposure disrupted male reproductive morphology, function, and endocrine regulation [24,31,49]. Oral exposure of IMI in male rats or mice altered sexual behavior, including increased latency in mount, intromission, and ejaculation while reducing their frequencies [47]; reduced body weight and reproductive organ weight, decreased epididymal sperm concentration, lower sperm motility, abnormal sperm morphology, increased apoptosis of cells in the seminiferous tubules and sperm cell DNA fragmentation, increased testicular fatty acid concentration, lower antioxidant levels, lower testosterone, and histopathological alterations in testis and epididymis [45,47,50,51,52,53,92]; reduced CYP3A4 enzyme activity [52]; altered serum and testicular cholesterol species [53]; and altered gonadotropic and gonadal hormones such as follicle stimulating hormone, testosterone, estradiol, and prolactin [47,50,51,53]. Additionally, IMI exposure induced changes in testicular gene expression, such as the downregulation of steroidogenic genes including *NR5A1* and *3b-HSD* [92] and *StAR*, *PBR*, *P450scc*, *3b-HSD*, *CYP19A1*, and *P450 17a* [47,53], reduction in testicular AR mRNA and protein [53], and upregulation of genes involved in the DNA damage tolerance (*OGG1*) [92]. Further, IMI treatment in rats increased the activities of sperm enzymes gamma-glutamyl transpeptidase, lactate dehydrogenase-x, and sorbitol dehydrogenase; decreased the activities of testicular enzymes 3b-HSD and 17b-hydroxysteroid dehydrogenase (17b-HSD); increased testicular lipid peroxidase levels; and reduced testicular glutathione (GSH), catalase (CAT), superoxide dismutase (SOD), glutathione peroxidase (GPx), and glutathione-S-transferase (GST) activity [51]. Neonatal IMI exposure in rats reduced antioxidant enzyme levels (CAT and SOD), reduced plasma testosterone, impaired spermatogenesis, and induced histological changes in the seminiferous tubules and epididymis [93]. Studies in rats reported that IMI exposure induced degeneration and necrosis of spermatocytes in the seminiferous tubules, intertubular edema, and hyperemia in the testes [94]. In addition, higher 8-hydroxy-2′-deoxyguanosine levels, indicative of oxidative stress, and higher caspase-3 expression, indicative of apoptosis, were observed in the testicular tissue. In rabbits, oral exposure to IMI caused genotoxic effects in lymphocytes, including a marked increase in binucleated cells with micronuclei and DNA damage within the micronuclei, as demonstrated by comet assays [27,29]. Metabolic analysis identified the aldehyde oxidase pathway as the major IMI biotransformation in male rabbits, supported by attenuated effects following co-treatment with sodium tungstate dihydrate (an aldehyde oxidase inhibitor) [29]. Moreover, feeding of IMI-sprayed grass to male rabbits induced severe testicular alterations, including thinning of the tunica albuginea, disorganized seminiferous tubules with increased lumen diameter, disorganized spermatogenic cells that were detached from the basement membrane, a decrease in the number of spermatogenic and Leydig cells, and an increased number of abnormal sperm [48].

The adverse effects of ACE on the reproductive system of rats and mice have been recently reviewed [31]. Treatment of mice with ACE resulted in reduced body weight, disorganized seminiferous epithelium, reduced expression of testis cell proliferation markers, *Ki67* and *Top2a,* and downregulation of *Lhr* in testis tissue [95], as well as decreased testicular weight and increased oxidative stress [96]. In rats and mice, ACE exposure altered seminiferous tubule diameters, decreased sperm concentrations, decreased sperm motility and viability, reduced plasma testosterone, and increased plasma LH levels [96,97,98,99,100]. In addition, GSH depletion and lipid peroxidation in the testes, mitochondrial damage and oxidative stress in Leydig cells, decreased synthesis of cAMP and ATP in the testes, reduction in testis expression of *StAR*, *Cyp11a1*, *Cyp17a1*, *Hsd17b1*, and *3-β-Hsd*, and disorganization of seminiferous tubules were reported [95,98,101]. ACE exposure in rats resulted in the degeneration and necrosis of spermatocytes, intertubular edema, and hyperemia in the testes [94]. In addition, higher 8-hydroxy-2′-deoxyguanosine levels, indicative of oxidative stress, and higher caspase-3 expression, indicative of apoptosis, were observed in the testicular tissue. In male guinea pigs, ACE impaired testicular structure, testosterone concentration, sexual behavior, and epididymal sperm count, motility, and plasma membrane integrity [102]. In zebrafish, ACE reduced survival rates, induced feminization, increased estradiol, and decreased androstenedione [46]. In addition, in male zebrafish, downregulation of the expression of steroidogenic genes such as *AR*, *CYP19b*, *FSHβ*, *GnRH2*, *GnRH3*, and *LHβ* in the brain and gonads was reported. The F1 generation embryos experienced decreased hatchability and malformation. Field-relevant ACE exposure of wild male house sparrows in captivity reduced their sperm density and caused a decrease in SOD enzymes, indicating potential fertility risks in wildlife [103].

Clothianidin (CLO) exposure in rats resulted in reduced weights of the right cauda epididymis and seminal vesicles [104,105]; decreased body weight, epididymal sperm concentrations, testosterone levels, and GSH levels [104]; increased sperm abnormalities, testis germinal epithelium TUNEL positive cells, and sperm DNA fragmentation [104]; and elevated testicular cholesterol, palmitic, linoleic, and arachidonic acids [104,105]. Chronically stressed male mice exposed to CLO demonstrated vacuolated seminiferous epithelium and decreased testicular antioxidant enzyme GPx, indicating exacerbated adverse reproductive damage under stress [106]. Prenatal and early postnatal CLO exposure in male mice reduced testis weight and germ cell numbers in seminiferous tubules, along with the appearance of germ cell-depleted tubules [107]. Young and adult male Japanese Crested Ibis quail exposed to CLO showed decreased germ cell numbers, increased vacuolization, and DNA fragmentation in the seminiferous tubules [108,109]. In addition, breeding of CLO-exposed male quails resulted in an increased rate of egg development failure, shorter embryo length, and lower embryo weight [108]. CLO exposure also significantly reduced testicular antioxidant GPx and SOD activities [109]. In honeybees, CLO exposure alters SOD activity in seminal fluid and GPx and CAT activities in spermatozoa, along with increased MDA and reduced seminal protein content, indicating impaired reproductive capacity [110].

Thiacloprid (THI) exposure causes significant reproductive toxicity in male mice. Exposure of immature male mice to THI altered sexual behavior, decreased spermatogenic cell layers, disrupted seminiferous epithelium organization, increased abnormal sperms, and downregulated mRNA of key spermatogenesis-related genes, including *Ki67*, *Ddx4*, *Atg5*, *Scp3*, and *Crem* [111]. In addition, THI exposure reduced testosterone and FSH levels and decreased *StAR* and *Cyp11a1* expression, thus affecting testosterone biosynthesis. Transgenerational studies revealed that male offspring of THI-exposed pregnant female mice exhibited reduced sperm counts, telomere defects, increased meiotic abnormalities, and dysregulation of testicular genes involved in translation, ATP production, and chromatin modification [112]. In addition, a higher number of DMC1 foci in sex chromosomes indicates persistence of breaks and telomere defects, such as formation of ring sex chromosomes, telomere end-to-end connections, synapsing defects, and telomere connection defects, along with altered expression of a major portion of the transcriptome, indicating global changes in protein translation. Furthermore, gestational exposure of male embryos to THI resulted in heritable epigenetic alterations in the F3 generation of male progeny, including abnormal DNA methylation, increased incidence of meiotic double-stranded breaks, incomplete synapsed chromosomes, and sperm-associated differentially methylated regions at the promoters of germ cell reprogramming-responsive genes [113].

The treatment with TXM in rats reduced serum testosterone levels and suppressed testicular GSH, SOD, and CAT activities, along with marked testicular histomorphological alterations [114,115]. Additionally, TXM exposure downregulated mRNA expression of *StAR*, *CYP17a*, *3β-HSD*, *SR-B1*, and *P450scc* genes in testicular tissue [114]. Functionally, these molecular and biochemical disruptions were associated with reduced sperm count, motility, and viability, along with increased incidence of abnormal sperm morphology [115], indicating impaired spermatogenesis and steroidogenesis.

Recently, chronic DINO exposure in rats impaired reproductive performance, evidenced by reduced mating and fertility indices, decreased testicular weight, poor sperm quality, and increased sperm aneuploidy [116]. These effects were accompanied by hormonal disruption, severe testicular histopathological damage, and elevated caspase-3 expression, indicating enhanced apoptosis. The details of additional studies on THI and TXM are provided in Supplementary Table S1 [117,118,119].

Taken together, our findings and interpretation on in silico neonicotinoid–AR interactions are consistent with existing experimental and epidemiological evidence linking neonicotinoid exposure to male reproductive toxicity. The results suggested that neonicotinoids may interfere with androgen signaling by perturbing testosterone binding to the AR, providing a plausible mechanistic basis for reported reproductive dysfunction. Although human data remain limited, available epidemiological studies support this interpretation. As this is a novel study, it warrants further validation through targeted in vitro and in vivo studies. Moreover, these findings may aid in the development of predictive screening tools for future toxicological and epidemiological assessments and support the rational design of neonicotinoid compounds with reduced risks to human health and the environment.

## 5. Conclusions

The present study investigated the structural interactions of eight neonicotinoid ligands, IMI, ACE, CLO, TXM, DIN, THI, NIT, and TTNM, against AR. In addition, MD simulation of a representative top-scoring neonictinoid IMI was performed to assess the stability of docking interactions. All ligands, except THI, exhibited favorable binding interactions with AR, as reflected by their binding free energies and binding affinities, with IMI showing the strongest interaction. A high degree of overlap was observed between AR-interacting residues of the native ligand testosterone and those of neonicotinoids ACE, CLO, TXM, and NIT (complete matching), and IMI and DNT (all but one residue matching). Hydrogen bonding, hydrophobic pi-alkyl interactions, and conserved residue contacts within the AR ligand binding domain further supported stable binding. MD simulation of AR–IMI complex demonstrated conformational stability, as evidenced by stable RMSD and RMSF profiles, reduced flexibility, increased compactness (Rg), minimal SASA changes, consistent hydrogen bond dynamics, and a stable free energy landscape. Collectively, these findings indicate that neonicotinoids can stably occupy the AR ligand-binding domain similarly to testosterone, suggesting that neonicotinoid exposure may disrupt androgen signaling and potentially impair male reproductive function. Given the predictive nature of this study, the findings warrant further validation through well-designed in vitro and in vivo experiments, as well as carefully controlled epidemiological and environmental investigations.

## Figures and Tables

**Table 1:** Compound names, common abbreviations, Chemical Abstract Service Registry Number (CAS No.), and PubChem IDs of neonicotinoid insecticide compounds.

Serial No.	Compound Name	Abbreviation	CAS No.	PubChem ID.
1	Imidacloprid	IMI	138261-41-3	86287518
2	Acetamiprid	ACE	135410-20-7	213021
3	Clothianidin	CLO	210880-92-5	86287519
4	Thiamethoxam	TXM	153719-23-4	5485188
5	Dinotefuran	DIN	165252-70-0	197701
6	Thiacloprid	THI	111988-49-9	115224
7	Nitenpyram	NIT	150824-47-8	3034287
8	Nithiazine	TTNM	58842-20-9	42853

**Table 2:** Comparative analysis of amino-acid residues and type of interactions for the native interaction pose and re-docking pose of the androgen receptor and testosterone. For both interaction poses, identical interacting amino-acid residues and types of interactions were observed.

Serial No.	Native Ligand Interaction		Re-Dock Ligand Interaction	
	Amino-Acid Residues	Interactions	Amino-Acid Residues	Interactions
1	Leu-704	Pi-alkyl	Leu-704	Pi-alkyl
2	Asn-705	H-bond	Asn-705	H-bond
3	Gln-711	H-bond	Gln-711	H-bond
4	Trp-741	Pi-alkyl	Trp-741	Pi-alkyl
5	Met-742	Pi-alkyl	Met-742	Pi-alkyl
6	Met-745	Pi-alkyl	Met-745	Pi-alkyl
7	Arg-752	H-bond	Arg-752	H-bond
8	Met-780	Pi-alkyl	Met-780	Pi-alkyl
9	Leu-873	Pi-alkyl	Leu-873	Pi-alkyl
10	Thr-877	H-bond	Thr-877	H-bond

**Table 3:** The binding strength scores, i.e., binding energy, binding affinity (pKi), and ligand efficiency for neonicotinoid ligands and testosterone in the ligand binding pocket of the androgen receptor during molecular docking. Thiacloprid did not bind in the same ligand binding site and did not show any interactions with the androgen receptor. Therefore, further analysis was not performed for any other parameters.

Serial No.	Ligand	Binding Free Energy (kcal/mol)	Binding Affinity pKi	Ligand Efficiency (kcal/mol/non-H Atom)
1	Testosterone	−11.5	8.43	0.479
2	Imidacloprid	−8.0	5.87	0.333
3	Acetamiprid	−7.1	5.21	0.323
4	Clothianidin	−6.6	4.84	0.275
5	Thiamethoxam	−7.3	5.35	0.292
6	Dinotefuran	−6.3	4.62	0.286
7	Thiacloprid	No analysis was performed		
8	Nitenpyram	−7.5	5.50	0.279
9	Nithiazine	−5.3	3.89	0.408

**Table 4:** Detailed list of molecular interactions, including number of bonds, interacting amino-acid residues, and distance, category, and type of interactions for neonicotinoid ligands and testosterone during molecular docking in the ligand binding pocket of the androgen receptor.

Ligand	Number of Bonds	Interacting Residues	Distance (Å)	Category	Type of Interaction
Testosterone	Total-10 H-bond-04 Pi-alkyl-06	Leu-704 Asn-705 Gln-711 Trp-741 Met-742 Met-745 Arg-752 Met-780 Leu-873 Thr-877	4.82 2.10 3.32 5.18 5.03; 5.35 3.88 2.96 5.02 4.31 2.99	Pi-alkyl H-bond H-bond Pi-alkyl Pi-alkyl Pi-alkyl H-bond Pi-alkyl Pi-alkyl H-bond	Hydrophobic Conventional Conventional Hydrophobic Hydrophobic Hydrophobic Conventional Hydrophobic Hydrophobic Conventional
Imidacloprid	Total-06 H-bond-05 Carbon H-bond-01	Leu-704 Asn-705 Gln-711 Met-745 Arg-752 Phe-764	3.56 2.45 3.00 3.28 2.86 3.16	Carbon H-bond H-bond H-bond H-bond H-bond H-bond	Conventional Conventional Conventional Conventional Conventional Conventional
Acetamiprid	Total-03 H-bond-02 Carbon H-bond-01	Leu-704 Arg-752 Thr-877	3.33 3.14 2.22	Carbon H-bond H-bond H-bond	Conventional Conventional Conventional
Clothianidin	Total-04 H-bond-02 Pi-alkyl-01 Pi-sulfur-01	Leu-704 Asn-705 Gln-711 Arg-752 Met-780	4.64 2.53 2.94 5.16 7.72	Pi-alkyl H-bond H-bond Unfavorable++ Pi-sulfur	Hydrophobic Conventional Conventional Unfavorable Miscellaneous
Thiamethoxam	Total-04 H-bond-02 Carbon H-bond-01 Pi-sulfur-01	Leu-704 Met-745 Met-780 Thr-877	3.16 3.56 5.75 2.13	H-bond Carbon H-bond Pi-sulfur H-bond	Conventional Conventional Miscellaneous Conventional
Dinotefuran	03 H-bond-03	Leu-704 Gly-708 Met-745 Arg-752	3.01 3.22 3.69 5.32	H-bond H-bond H-bond Unfavorable++	Conventional Conventional Conventional Unfavorable
Thiacloprid	No interactions				
Nitenpyram	Total-02 H-bond-02	Asn-705 Arg-752	2.10 3.33; 2.96	H-bond H-bond	Conventional Conventional
Nithiazine	Total-08 H-bond-02 Pi-alkyl-04 Pi-charge-02	Glu-681 Gln-711 Val-715 Trp-718 Leu-744 Met-745 Ala-748 Arg-752	4.62 3.70 4.06 4.81 4.67 4.39 4.40 2.72	Pi-charge H-bond Pi-alkyl Pi-charge Pi-alkyl Pi-alkyl Pi-alkyl H-bond	Electrostatic Conventional Hydrophobic Electrostatic Hydrophobic Hydrophobic Hydrophobic Conventional

**Table 5:** Time-averaged molecular dynamics simulation parameters showing the data for the structural and energetic stability of the native androgen receptor (AR) and the AR–imidacloprid (AR–IMI) complex during the 100 ns simulation. Compared with native AR, the AR–IMI complex exhibits lower potential energy, reduced mean square deviation, slightly decreased radius of gyration and solvent accessible surface area, and a marginally higher hydrogen-bond count, collectively indicating enhanced structural stability and compactness of AR upon IMI binding.

Endpoints	Simulated Systems	
	Androgen Receptor	Androgen Receptor-IMI Complex
Average potential energy (kJ/mol)	−700,027.540	−713,825.670
Root mean square deviation (nm)	0.281	0.252
Radius of gyration (nm)	1.740	1.723
Solvent accessible surface area (nm^2^)	130.302	129.162
Hydrogen bond dynamics (number)	198	201

## Data Availability

The majority of the data for the results of this study are provided in the main manuscript. In addition, any specific data will be made available on request to the corresponding author.

## Supplementary Materials

The following supporting information can be downloaded at: https://www.mdpi.com/article/10.3390/biology15020126/s1, Table S1: Reproductive effects of various neonicotinoid compounds on different species.
