# Supplementary material for: Neonicotinoids and the Androgen Receptor: Structural Dynamics and Potential Signaling Disruption
Source: Biology (Basel). 2026 Jan 10;15(2):126. doi: 10.3390/biology15020126 (PMC12837828; doi:10.3390/biology15020126)
Supplement: Supplementary file 1 [file biology-15-00126-s001.zip › biology-4006392-supplementary.pdf]

**Supplementary Table S1**

Reproductive effects of various neonicotinoid compounds on different species. Results of *in silico* docking interactions and molecular dynamics (MD) simulation in the present study and one available previous study are also given.

| **Neonicotinoid compound** | **Interactions with androgen receptor (*in silico*)** | **Reproductive effects i*n vivo;* (species and sample)** | **Brief details of effects on reproductive system** | **Reference** |
|---|---|---|---|---|
| Imidacloprid (IMI) | IMI bound (docking) to the ligand binding domain of human androgen receptor (PDB ID 2AM9); the interaction complex involved six amino-acid residues (Leu-704, Asn-705, Gln-711, Met-745, Arg-752, Phe-764) and six hydrogen bonds; the dynamic stability of the | Human (blood, semen) | Low sperm count, low sperm motility and abnormal sperms; negative correlation between sperm concentration and motility, and seminal IMI and its metabolite (6-chloronicotinic acid) | [50] |
| | | Human (urine, serum) | Positive association with serum testosterone, dehydrocorticosterone, and dehydroepiandrosterone; positive association of IMI metabolite (IMI-olefin) and DHEA | [89] |
| | | Human (semen) | Lower total and progressive sperm motility associated with IMI-olefin | [40] |
| | | Human (urine, serum) | Serum total testosterone 21% lower with 10-fold increase in urinary 5-hydroxy-imidacloprid (IMI metabolite) | [91] |
| | | Rat (serum, testis) | Reduced body weight, reduced weight of epididymis, cauda epididymis, and seminal vesicles; higher abnormal sperm rate; increased apoptotic indexes in testis and increased sperm DNA fragmentation; reduced serum testosterone; higher malondialdehyde (MDA) and lower glutathione (GSH) in testis tissue | [45] |
| | | Rat (serum, testis) | Decreased bodyweight and epididymal weigh; decreased total epididymal sperm count, sperm motility, and live sperm count; | [51] |

| | | | | |
|---|---|---|---|---|
| | complex confirmed by MD simulation (present study) | | increased sperm abnormalities; increased activities of gamma-glutamyl transpeptidase (g-GT), lactate dehydrogenase-x (LDH-x), and sorbitol dehydrogenase (SDH); 3β-HSD and 17β-HSD enzymatic activity deceased, testosterone concentration in testis and plasma decreased; increase in lipid peroxidation (LPO) and decrease in GSH, decreased activities of catalase (CAT), superoxide dismutase (SOD), glutathione peroxidase (GPx), and glutathione-S-transferase (GST); histopathological alterations in testis and epididymis. | |
| | IMI docked into the active site of mouse androgen receptor (PDB ID: 2QPY) successfully; the interaction complex showed hydrogen bonds and several amino-acid residues in the ligand-binding pocket of the AR; The dynamic stability of the complex was confirmed by MD simulation [53] | Rat (serum, testis) | Reduction in body weight, testis and seminal vesicle weight; decreased sperm concentrations and increased sperm abnormalities; decrease serum testosterone; downregulation of steroidogenic genes, *nuclear receptor subfamily 5 group A member 1* (*NR5A1) and 3-beta-hydroxysteroid dehyrodrogenase (3b-HSD)* and upregulation DNA damage tolerance gene (*OGG1*); increased serum 8-hydroxy-2′-deoxyguanosine; increased apoptosis of spermatogonial cells | [92] |
| | | Rat (serum, testis) | Decreased sperm concentrations, increased sperm abnormalities; lowered testosterone; histopathological changes in testis; decreased cytochrome P450 3A4 activity | [52] |
| | | Rat (serum, testis) | Decreased territorial aggression; increase in latency in mount, intromission and ejaculation; reduced frequencies of mounts, | [47] |

| | | | | |
|---|---|---|---|---|
| | | | intromission, and ejaculation; prolonged post-ejaculatory intervals and decreased intromission ratio; decrease serum testosterone along with increase FSH and LH; disturbed redox state (increased MDA, reduced GSH content and CAT activity in testicular tissues); decreased sperm motility, concentration, and viability with increased sperm abnormalities; reduced testicular expression of *StAR* and aromatase genes; severe degeneration and necrosis in both germ cells and Leydig cells with atrophy of seminiferous tubules | |
| | | Rat (testis) | Severe degeneration and necrosis in the spermatocytes at the tubular wall; severe edema in the tubular region and thinning of tubular wall; severe intracytoplasmic 8-OHdG and caspase-3 expressions in testis tissue indicating apoptosis; reduced sperm cell densities | [94] |
| | | Rat (plasma, testes) | Reduced body weight and testicular weight; decrease in testis tissue CAT and SOD activity, increase in testis tissue reactive oxygen species; decrease in plasma testosterone level; reduced number of spermatocytes with arrested spermatogenesis; decrease in seminiferous tubular diameter, increase in tubular lumen diameter, and decrease in tubular epithelial height; degeneration of epididymal tubules with increase in the interstitial space and reduction in the stromal space surrounding the lumen | [93] |

| | | | | |
|---|---|---|---|---|
| | | Mice (serum, testis) | Decreased testis weight; histological damage including thinner seminiferous tubules, decreased layers of spermatogenic cells, and irregular seminiferous epithelium; decreased serum testosterone and estradiol levels and aromatase activity; decreased androgen receptor mRNA expression and protein immunostaining in testis | [53] |
| | | Rabbit (testis) | Thinning of the tunica albuginea of the testes; disorganized seminiferous tubules with increased lumen diameter; disorganized spermatogenic cells detached from the basement membrane; decreased number of spermatogenic and Leydig cells; increased number of abnormal sperm | [48] |
| | | Lizard (serum, testis) (wild caught and exposed under laboratory and simulated environment) | Death in acute high dose; sub-chronic doses cause arrest of spermatogenesis, multinucleated cells, narrow tubular lumen, reduced interstitial tissue, and degeneration at higher dose; increased apoptosis; reduced serum testosterone and estradiol; decreased expression of mRNAs for androgen and estrogen receptors in testis tissue; similar results under simulated environment. | [16] |
| **Acetamiprid (ACE)** | | Human (urine, serum) | Positive association of urinary N-desmethyl-ACE with serum androstenedione levels | [89] |
| | | Human (urine, serum) | Total serum testosterone lower by 25% with 10-fold increase in urinary N-desmethyl-ACE (ACE metabolite); urinary detection of N-desmethyl-ACE associated with 21% lower serum testosterone; free androgen index was | [91] |

| | | | | |
|---|---|---|---|---|
| | ACE bound (docking) to the ligand binding domain of human androgen receptor (PDB ID 2AM9); the interaction complex involved three amino-acid residues (Leu-704, Arg-752, Thr-877) and three hydrogen bonds (present study) | | lower by 23% with 10-foldincrease in N-desmethyl-acetamiprid | |
| | | Rat (plasma, testis) | Plasma testosterone concentration and sperm quality decreased; plasma LH increased; increased MDA and nitric oxide (NO) and reduced adenosine triphosphate (ATP) and cyclic adenosine monophosphate (cAMP) in Leydig cells; decreased protein levels of StAR, hydroxy-delta-5-steroid dehydrogenase, HSD3B, and cytochrome P450, family 11, subfamily a, polypeptide 1 (CYP11A1); decreased testicular mRNA levels; mitochondrial membrane damage in Leydig cells; increased oxidative stress in Leydig cells; reduced testicular ATP and cAMP. | [98] |
| | | Rats (serum, testis) | Sperm count decreased; sperm abnormalities increased; plasma testosterone and cholesterol decreased; plasma LH, GnRH, and inhibin B increased; testis tissue and plasma GSH and total antioxidant status decreased while MDA and total oxidant status (TOS) increased; vacuole formation in germinal epithelium, immature cells in tubules, decreased number of spermatogenic germ cells, damaged spermatogenic cells, irregular and undulating basement membrane, seminiferous tubule score decreased, proliferative cell nucleus antigen (PCNA)-positive cells observed in seminiferous tubules, proliferation index decreased, apoptotic index increased | [99] |

| | | | | |
|---|---|---|---|---|
| | | Rat (serum, testis) | Induced large basal vacuoles in Sertoil cells and disorganized stratification of spermatogonia; decreased sperm motility and viability, and increased abnormal sperms; caused acrosome damage and DNA fragmentation; increased thiobarbituric acid reactive substances (TBARS), decreased GSH, GPx, and glutathione reductase (GR), decreased GST, increased CAT; decreased plasma testosterone | [101] |
| | | Rat (serum, testis) | Decrease in the sperm count, viability and motility; decreased plasma testosterone and gonadotropin-releasing hormones (GnRH) | [100] |
| | | Rat (testis) | Severe degeneration and necrosis in the spermatocytes at the tubular wall; severe edema in the tubular region and thinning of tubular wall; severe intracytoplasmic 8-OHdG and caspase-3 expressions in testis tissue indicating apoptosis; reduced sperm cell densities | [94] |
| | | Mice (serum, testis) | Degeneration in seminiferous tubules, increased tubule lumen; decreased serum testosterone; synergistic adverse effect on the testis with glyphosate | [97] |
| | | Mice (serum, testis) | Decreased body weight; decreased weight of the testis, epididymis, and seminal vesicles; decreased sperm count, viability and motility; increased acrosome deformity; decreased serum testosterone; vacuolization of seminiferous tubules, and decreased spermatids and interstitial Leydig cells with sloughing of the tubular lumen; primary | [96] |

| | | | | |
|---|---|---|---|---|
| | | | spermatocytes vacuolized and interstitium widened; no sperm in seminiferous tubule lumen; mitochondria were swollen; increased MDA and NO; decreased activity of CAT, GSH-Px and T-SOD | |
| | | Mice (serum, testis) | Decreased body weights; abnormal seminiferous epithelium; lower expression of cell proliferation markers Ki67 and Top2a; downregulation of testis mRNA expression of *LHr, StAR, Cyp11a1, Cyp17a1*, *and Hsd17b1* genes | [95] |
| | | Guinea Pig (serum, testis) | Reduced weight of testes, epididymis, vas deferens, and supporting glands; adversely affected testicular structure; reduced reaction time; reduced testosterone concentration; reduced sperm count, motility, and plasma membrane integrity; increased abnormal spermatozoa; concentration of MDA and activities CAT and SOD increased; accumulation of non-differentiated cells in tubule lumen and interstitial tissue markedly destroyed | [102] |
| | | House sparrow (semen) (wild caught and exposed in laboratory) | Reduced sperm density and decreased activity of sperm SOD activity | [103] |
| | | Zebra fish (testis) | Reduced survival, induced feminization, body length and body weight increased; decreased brain somatic index, hepatosomatic index and gonadosomatic index; male gonad development inhibited; increased E2 and decreased androstenedione; downregulation of genes (*gnrh2, gnrh3, ar, cyp19b, fshβ, and* | [46] |

| | | | | |
|---|---|---|---|---|
| | | | *lhβ)* in brain; upregulation of genes (*fshr, 3βhsd, cyp11a, cyp17, lhr, and cyp19a*) in testis; adverse effects on F0 generation; transgenerational effects | |
| **Clothianidine (CLO)** | CLO bound (docking) to the ligand binding domain of human androgen receptor (PDB ID 2AM9); the interaction complex involved five amino-acid residues (Leu-704, Asn-705, Gln-711, Arg-752, Met-780), two hydrogen bonds, one pi-alkyl, and one pi-sulfur bond (present study) | Human (urine, serum) | Positive association with serum androstenedione; negative association with cortisone | [89] |
| | | Rat (serum, testis) | Decreased body weight and weights of cauda epididymis and seminal vesicles; decreased sperm concentration and increased abnormal sperm rates; decreased serum testosterone; decrease in GSH, increase in apoptosis in germinal epithelium; elevated docosapentaenoic, arachidonic, palmitic and palmitoleic acids, increased sperm DNA fragmentation | [104] |
| | | Rat (serum, testis) | Decreased weights of epididymis, right cauda epididymis and seminal vesicles; increased apoptosis and increased level of TBARS and cholesterol, and elevated palmitic, linoleic and arachidonic acids in testis | [105] |
| | | Mice (testis) | Decreased body weight; enhanced anxiety-like behavior; vacuolizations in seminiferous tubules and seriously degenerated seminiferous tubules with no germ cells; abnormal immunoreactivity of GPx4 in Sertoli cells | [106] |
| | | Mice (testis) | Reduced testis weight; reduced number of germ cells in seminiferous tubule | [107] |
| | | Quails (testis, embryos) | Neurobehavioral problems (convulsions, tottering, feather ruffling and crouching); increased body weight; vacuolization in the | [108] |

| | | | | |
|---|---|---|---|---|
| | | | seminiferous epithelia, decreased number of germ cells, germ cell DNA fragmentation, and greater number of ssDNA positive cells in seminiferous tubules; shorter size of embryos | |
| | | Honey bee (semen) | Increased SOD activity in seminal fluid; increased GPx and CAT activities in spermatozoa; increased MDA and reduced protein content in semen | [110] |
| **Thiacloprid (THI)** | THI did not bind (docking) to the androgen receptor ligand binding domain in the present study | Human (urine) (children) | Positive correlation with delayed genitalia development in boys and positive correlation with early axillary hair development in girls | [90] |
| | | Rat (serum, testis) | Reduced body weight and testis weight; reduced sperm count, motility and viability, and increased sperm abnormalities; decreased serum testosterone and increased FSH and LH; decline in testis 3βHSD and 17βHSD activity; elevated testis MDA, and reduced activities of testis GSH, CAT and SOD; degenerative changes in seminiferous tubules, apoptotic spermatogenic cells, decreased epithelial height, interstitial tissue with high accumulation of fibrous connective tissue, and vacuoles in Leydig cells; testicular cell DNA damage | [117] |
| | | Rat (serum, testis) | Decreased body weight; increased testis to body weights ratios; decreased sperm count; increase in cell number in testis tubules; decrease in tubule lumen; vacuoles in tubular cells; expression of spermatocyte marker genes *Rad51 and Hormad1* increased; increase in testis cell density; higher number | [112] |

| | | | | |
|---|---|---|---|---|
| | | | of DMC1 foci in sex chromosomes indicating persistence of breaks; telomere defects such as formation of ring sex chromosomes and telomere end-to-end connections, increase in the number of synapsing defects; telomere connection defects; 598 differential transcripts corresponding to 560 genes were deregulated; global change in the expression of protein translation, ATP-dependent and chromatin-modifying genes; alteration of chromatin structure potentially leading to changes in meiotic progression and gene expression | |
| | | Mice (serum, testis) | Diminished sexual behavior, decrease in spermatogenic cell layers and irregular seminiferous epithelium; increased sperm abnormalities; down-regulation of mRNA levels of spermatogenesis-related genes *Ddx4*, *Scp3, Atg5*, *Crem, Ki67*, in testis tissue; reduced serum testosterone and FSH; decreased expression of steroidogenesis genes *Star* and *Cyp11a1* in testis tissue | [111] |
| **Thiamethoxam (TXM)** | TXM bound (docking) to the ligand binding domain of human androgen receptor (PDB ID 2AM9); the interaction complex involved five amino-acid residues (Leu-704, | Human (urine, serum) | Positive association with serum androstenedione, and negative associations with serum cortisone, dehydrocorticosterone, and deoxycorticosterone levels | [89] |
| | | Rat (serum, testis) | Decline in serum testosterone, reduction in SOD, CAT, and GSH enzyme activities, and elevated testis MDA and NO level; downregulation of mRNA expression of testicular steroidogenic genes (*StAR, CYP17a, 3β-HSD, SR-B1, and P450scc)* and | [114] |

| | | | | |
|---|---|---|---|---|
| | Met-745, Met-780, Thr-877), three hydrogen bonds, and one pi-sulfur bond (present study) | | upregulation of *LHR and aromatase genes*; diffuse testicular degeneration with irregular, disorganized seminiferous tubules showing lumen with detached germ cells and absence of luminal mature sperm; interstitium showed hyalinization of interstitial connective tissue; sometimes tubules with testicular necrosis and without tubular lumen | |
| | | Rat (serum, testis) | Decreased body weight and increased testis weight; reduced sperm count, viability, motility, and progressive motility; increase in semen fructose level and sperm abnormalities; reduced serum testosterone levels and increased FSH and LH; elevated testis tissue MDA and reduction in GSH and CAT activities; disruption of seminiferous tubules with reduced spermatogenic cell numbers; decrease in interstitial cells with enlargement of interstitial areas, vacuolation and vascular thrombosis, pyknotic or pigmented spermatogenic, and decrease in seminiferous tubule diameter | [115] |
| | | Mice (serum, testis) | Reduced sperm count and spermatogenesis; decreased serum testosterone; degenerative changes in seminiferous tubules, Leydig and Sertoli cells; few or no sperm in the epididymis and homogeneous material and cellular debris in lumen | [118] |
| | | Solitary bee (semen) | Sperm quantity, viability, total living sperm severely reduced | [119] |
| Dinotefuran | Molecular docking to many enzymes | Rat (serum, testis) | Impaired reproductive performance (reduced mating and fertility indices), decreased | [116] |

| | | | | |
|---|---|---|---|---|
| | and receptors AR and reported strong binding [116]. | | testicular weight, poor sperm quality, increased sperm aneuploidy, hormonal disruption, severe testicular histopathological damage, and enhanced apoptosis | |
| Reproductive effects-related studies on nitenpyram, and nithiazine on human or laboratory animal are not available | | | | |
